# Ginseng Extracts Restore High-Glucose Induced Vascular Dysfunctions by Altering Triglyceride Metabolism and Downregulation of Atherosclerosis-Related Genes

**DOI:** 10.1155/2013/797310

**Published:** 2013-09-30

**Authors:** Gabriel Hoi-huen Chan, Betty Yuen-kwan Law, John Man-tak Chu, Kevin Kin-man Yue, Zhi-hong Jiang, Chi-wai Lau, Yu Huang, Shun-wan Chan, Patrick Ying-kit Yue, Ricky Ngok-shun Wong

**Affiliations:** ^1^Department of Biology, Hong Kong Baptist University, Kowloon Tong, Kowloon, Hong Kong; ^2^School of Chinese Medicine, Hong Kong Baptist University, Kowloon Tong, Kowloon, Hong Kong; ^3^State Key Laboratory of Quality Research in Chinese Medicine, Macau University of Science and Technology, Avenida Wai Long, Taipa, Macau; ^4^School of Biomedical Science, The Chinese University of Hong Kong, Shatin, N.T., Hong Kong; ^5^Department of Applied Biology and Chemical Technology, The Hong Kong Polytechnic University, Hung Hom, Kowloon, Hong Kong

## Abstract

The king of herbs, *Panax ginseng*, has been used widely as a therapeutic agent vis-à-vis its active pharmacological and physiological effects. Based on Chinese pharmacopeia *Ben Cao Gang Mu* and various pieces of literature, *Panax ginseng* was believed to exert active vascular protective effects through its antiobesity and anti-inflammation properties. We investigated the vascular protective effects of ginseng by administrating ginseng extracts to rats after the induction of diabetes. We found that *Panax ginseng* can restore diabetes-induced impaired vasorelaxation and can reduce serum triglyceride but not cholesterol level in the diabetic rats. The ginseng extracts also suppressed the expression of atherosclerosis-related genes and altered the expression of lipid-related genes. The results provide evidence that *Panax ginseng* improves vascular dysfunction induced by diabetes and the protective effects may possibly be due to the downregulation of atherosclerosis-related genes and altered lipid metabolism, which help to restore normal endothelium functions.

## 1. Introduction

*Panax ginseng* is one of the most commonly used Chinese medicine and research targets. The major active components of *Panax ginseng* are ginsenosides which can be sub-divided into three groups according to their basic structures: protopanaxadiol (PPD) type (e.g., Rb1, Rb2, Rc, Rd, Rg3, and Rh2), protopanaxatriol (PPT) type (e.g., Re, Rf, Rg1, Rg2, and Rh1), and oleanolic acid (e.g., Ro). Ginsenosides appear to be responsible for most of the activities of ginseng including antioxidation, anti-inflammation, and anticancer [1]. A review by Karmazyn et al. has found that the yearly ginseng-related publication has been increasing exponentially from 1950 to 2010. They also reported that *Panax ginseng* played a protective role in the cardiovascular system [2]. This suggested the beneficial properties of ginseng on cardiovascular diseases in both experimental and clinical settings.

Atherosclerosis is one of the most common cardiovascular diseases and can remain asymptomatic for decades. In the mid 1970s, Russel Ross developed the popular “response to injury” theory by postulating that atherosclerosis begins with injuries on the endothelium, followed by adhesion and aggregation of platelets [3]. At about the same time, Robert F. Furchgott, the Nobel Prize Laureate in Physiology or Medicine in 1998, discovered that acetylcholine induces endothelium-dependent relaxation in normal aortic tissue [4]. Upon early onset of atherosclerosis, endothelium can remain morphologically intact though inflammatory responses are triggered. Since then, numerous researches have been conducted to investigate the mechanisms of atherosclerosis to mitigate the associated diseases including adhesion of lipid-laden macrophages and smooth muscle cells which could finally result in endothelial denudation [5]. Besides, Hansson's research groups have reported that high level of total cholesterol and low density lipoprotein accumulated in the intima of the arteries, with the attack of myeloperoxidase and lipoxygenases, or by reactive oxygen species [6, 7] could also cause the early onset of atherosclerosis.

The primary objective of this study is to evaluate the protective effects of *Panax ginseng* on diabetes mellitus, a pathological condition which links to endothelial dysfunctions, through investigating the physiological parameters such as blood glucose, blood cholesterol, insulin, and advanced glycation end product in diabetic rat models. Furthermore, the changes of atherosclerosis-related genes expression in diabetic rats are also investigated after ginseng administration. The findings may help in the development of successful therapeutic interventions for atherosclerotic cardiovascular disease.

## 2. Materials and Methods

This study follows “The International Guiding Principles for Biomedical Research Involving Animals,” The Hong Kong Code of Practice for Care and Use of Animals for Experimental Purposes (2004). All experimental procedures were conducted according to the Animals (Control of Experiments) Ordinance of the Department of Health, HKSAR (Animal Licenses ID: (11-6) DH/HA&P/8/2/6 Pt.2; (10-4) DH/HA&P/8/2/6 Pt.1; (10-9) DH/HA&P/8/2/6 Pt.1). All animal studies were performed in facilities approved by the Animal Ethics Committee of the Chinese University of Hong Kong (10/028/MIS).

### 2.1. Animals

Male Sprague-Dawley (SD) rats weighing 150–200 grams were housed in room under standard vivarium conditions with 12 hour light/dark cycle. Throughout the experimental period, animals were fed with standard rodent chow and water available *ad libitum*. The animals were acclimatized to the laboratory conditions for 10 days prior to the inception of experiments. Experimental diabetic condition was induced in rats by a single intraperitoneal injection (i.p.) of streptozotocin (75 mg/kg body weight) freshly dissolved in cold citrate buffer (0.1 M), while the normal control group was injected with citrate buffer only. Blood samples were collected from tail veins of overnight-fasted rats three days after streptozotocin administration. Rats with blood glucose level higher than 16.7 mmol/dL were selected for experiment. 

The experimental rats were divided into seven groups: (1) normal control rats administered with water, (2) diabetic group of rats administered with water, (3) diabetic group administered with intraperitoneal injection of insulin, (4) diabetic group fed with PPT-type of ginseng (10 mg/kg/day), (5) diabetic group fed with PPT-type of ginseng (30 mg/kg/day), (6) diabetic group fed with PPD-type of ginseng (10 mg/kg/day), and (7) diabetic group fed with PPD-type of ginseng (30 mg/kg/day). The dosage of insulin followed a protocol developed by Kuo et al. [8], and water or drugs were administered for a total of 14 consecutive treatment days. Both PPD and PPT were administered orally in the form of aqueous suspension. Rats were anaesthetized by Ketamine-Rompun mixture (7.5 : 1), and blood was collected from the heart for further analysis. The animals were then sacrificed immediately by cervical dislocation. Aortae were removed and trimmed for tissue bath experiment. Other rat tissues including brain, heart, liver, spleen, eye, kidney, and aorta were immediately removed and instantly soaked in liquid nitrogen and stored at −70°C for further biochemical analysis. 

### 2.2. Ginseng Preparation

**Figure 1:**
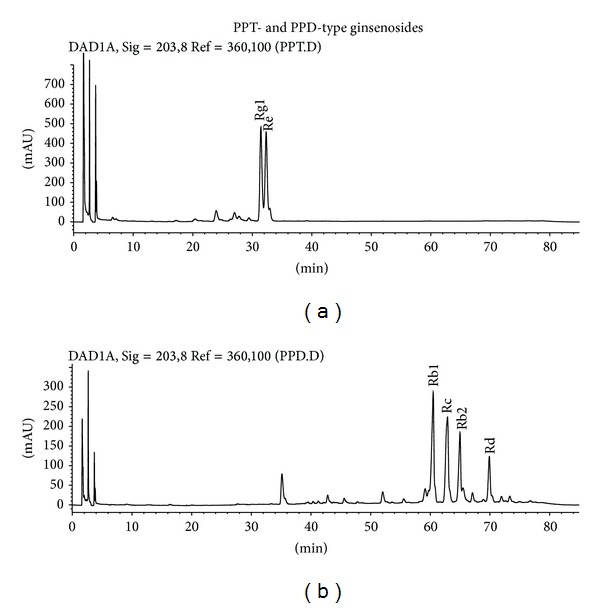
HPLC fingerprint of PPT-type (Panel (a)) and PPD-type (Panel (b)) ginseng extract used in current study. *Instruments.* An HP 1100 system (Hewlett-Packard, Wilmington, DE) consisting of a G1312A binary pump, a G1329A automatic sample injector, and a G1315A diode array detector was used to perform HPLC analysis. *Sample Preparation.* Approximately 0.20 g powdered ginseng was accurately weighed into a 50 mL conical flask, and 10 mL 70% methanol was added. The suspension was sonicated for 30 min, and the sample solution was filtered through a 0.45 *μ*m filter and used as the test solution for quantitative analysis of ginsenosides in Radix Ginseng. *Chromatographic Conditions.* HPLC analysis of Radix Ginseng was performed on an Alltima C_18_ HPLC column (4.6 mm × 250 mm, 5 *μ*m) at 25°C with a sample injection volume of 20 *μ*L. The mobile phase was a gradient elution of KH2PO4 buffer (2 mmol/L, pH 6.8) and acetonitrile, starting isocratically with 21% acetonitrile for 15 min and increasing to 38% acetonitrile over 55 min. The flow rate of the mobile phase was 1.0 mL/min, and the detector wavelength was 203 nm.

*Panax ginseng* extract was prepared as described in Zhu et al. [9], which meets the requirement of the Chinese Pharmacopoeia and Hong Kong Standard of Chinese Materia Medica. Standardized ginseng extract (RSE) was prepared by ethanol extraction. The residue was then dissolved in water and partitioned successively with petroleum ether, EtOAc, and n-BuOH to give the petroleum-ether-soluble, EtOAc-soluble, and n-BuOH-soluble fractions. The n-BuOH extract was subjected to column chromatography eluted with a CHCl_3_/MeOH gradient. Fractionated samples were combined and obtained according to the thin layer chromatography analysis. All samples were then stored in desiccated condition until further use. High performance liquid chromatography was used to confirm the identity of our samples with the standard ginsenosides (HPLC purity > 98%) purchased from Chengdu Scholar Bio-Tech Co. Ltd. (Chengdu, China) or National Institute for the Control of Pharmaceutical and Biological Products (Beijing, China). The contents of ginsenosides Rg1, Re, Rb1, Rc, Rb2, and Rd were 290.9, 339.6, 246.3, 231.3, 136.0, and 84.5 mg/g, respectively (Figure 1 and Table 1). 

### 2.3. Measurement of Contractile and Relaxant Responses in the Rat Aortic Rings

Similar procedures were followed according to the protocol as described by Chan and Fiscus 2002 [10]. Briefly, thoracic aortae were isolated by cutting from the aortic arch to the diaphragm, resulting in a length of 30–40 mm tissue. In order to prevent physical damage of endothelium by forceps, the parts from the aortic arch were not used for experiment. Fat tissues were trimmed off from the aortae and before it was cut into 3 mm segments rings. The segments were then mounted carefully between two platinum hooks in 10 mL organ baths containing Krebs buffer (KRB) maintained at 37°C bubbled with 95% O_2_–5% CO_2_ continuously. Following a 30 min equilibration period of resting tension of 1 gram, cumulative doses of phenylephrine (1 × 10^−9^ M to 1 × 10^−5^ M) were added in each aortic ring. After the addition of phenylephrine, the aortic rings were washed with fresh and bubbled KRB solution every 10 minutes over a 30-minute period. A single dose of phenylephrine at 1 × 10^−7^ M was added until the aortic rings maintained 50 percent of maximum tension. Doses of acetylcholine (1 × 10^−9^ M to 1 × 10^−5^ M) were added cumulatively to check the endothelial functions. All of the doses were added after the responses reached plateau.

### 2.4. Blood Profile of the Experimental Rats

Serum tests for total cholesterol (TC), triglyceride (TG), low density lipoprotein (LDL), and high density lipoprotein (HDL) were conducted in The State Key laboratory for Chinese Medicine and Molecular Pharmacology of the Hong Kong Polytechnic University. Terminal colorimetric analysis method was used for quantification of TC, TG, LDL, and HDL, respectively. The tests were conducted with ECHO automatic biochemistry analyzer (Logotech, Italy) and UV2800 spectrophotometer (Unico, Shanghai).

Blood glucose level was measured using glucometer (Elite, Bayer Corporation, USA). Serum insulin and glycation end products were measured using kits purchased from Millipore (EZRMI-13K) and Cusabio (CSB-E08140r), respectively.

### 2.5. *RT* ^2^ Profiler Rat Atherosclerosis PCR Array Analysis

Aortic samples for PCR array analysis were used to obtain the total RNA by Qiagen RNeasy Mini Kit (Catalogue number: PARN-038A, Qiagen). This pathway specific RT-PCR array was used to evaluate the potential alterations of related genes after PPD-type and PPT-type treatments (30 mg/kg/day) in rats. The atherosclerosis array comprised of 87 genes selected based on their involvement in regulating vascular and endothelial cell homeostasis or inflammation. There were 5 housekeeping genes served as positive controls. Total RNA was reverse transcripted using the RT^2^ First Strand Kit. Real-time PCR reactions were carried out on ABI 7500 (Applied Biosystems) using the RT^2^ SYBR Green qPCR Mastermix (Qiagen) according to manufacturer's instructions. Data analysis was performed using the Qiagen's integrated web-based software package for the PCR Array System, which automatically performs all ΔΔCt based fold-change calculations from raw threshold cycle data. 

### 2.6. Statistical Analysis

All values are expressed as mean ± standard error of mean (SEM). The significant differences between the young and aged groups in the isolated tissue experiments were analyzed using one way ANOVA with Newman-Keuls multiple comparison as post hoc test in the statistical package (Graphpad prism v6.0). A *P* value less than 0.05 was considered to be significant. The mean values were obtained from at least 5 animals or 3 DNA samples per treatment group.

## 3. Results

### 3.1. Ginseng Extracts Restore High Glucose-Induced Endothelial Dysfunction

**Figure 2:**
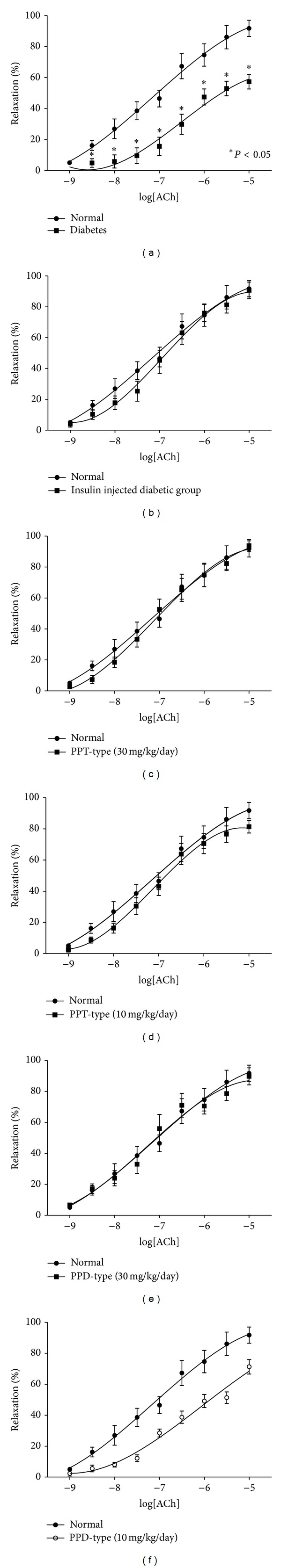
Ginseng extracts restore acetylcholine-induced endothelium dependent vasorelaxation. After the addition of phenylephrine, doses of acetylcholine (1 × 10^−9^ M to 1 × 10^−5^ M) were added cumulatively to check the endothelial functions. Control group showed an attenuation of acetylcholine-induced vasorelaxation (Panel (a)). The insulin injected diabetic group (Panel (b)), PPT-type (30 mg/kg/day) fed diabetic group (Panel (c)), PPT-type (10 mg/kg/day) fed diabetic group (Panel (d)), PPD-type (30 mg/kg/day) fed diabetic group (Panel (e)), and PPD-type (10 mg/kg/day) (Panel (f)) fed diabetic group showed restoration of the attenuated vasorelaxation. Results were expressed as the mean ± standard error; *P < 0.05 for the indicated comparisons.

Acetylcholine (ACh) causes vasodilation by activation of endothelial nitric oxide synthase and prostaglandin production. The aortic tissue was challenged with acetylcholine (1 × 10^−9^ M–1 × 10^−5^ M) and caused concentration-dependent relaxations in aortic rings from young rats. Normal rats showed 100% relaxation (restored the contracting state to resting state) at maximum dose 1 × 10^−5^ M, while the response was only 62.5% of the relaxation in the diabetic rats (Figure 2(a)), showing an impairment of the endothelium. For positive control, diabetic rats were injected with insulin and the normal vasorelaxation was maintained (Figure 2(b)). After feeding PPD-type and PPT-type ginseng extracts for two weeks, the impaired vasorelaxation due to high glucose level was restored (Figures 2(c) to 2(f)), indicating that the endothelial functions were maintained under the diabetic conditions for the ginseng-fed groups. 

### 3.2. Blood Profile, Body Weight, Distribution of Visceral Adipose Tissue, and Organs Weight of the Experimental Rats

**Figure 3:**
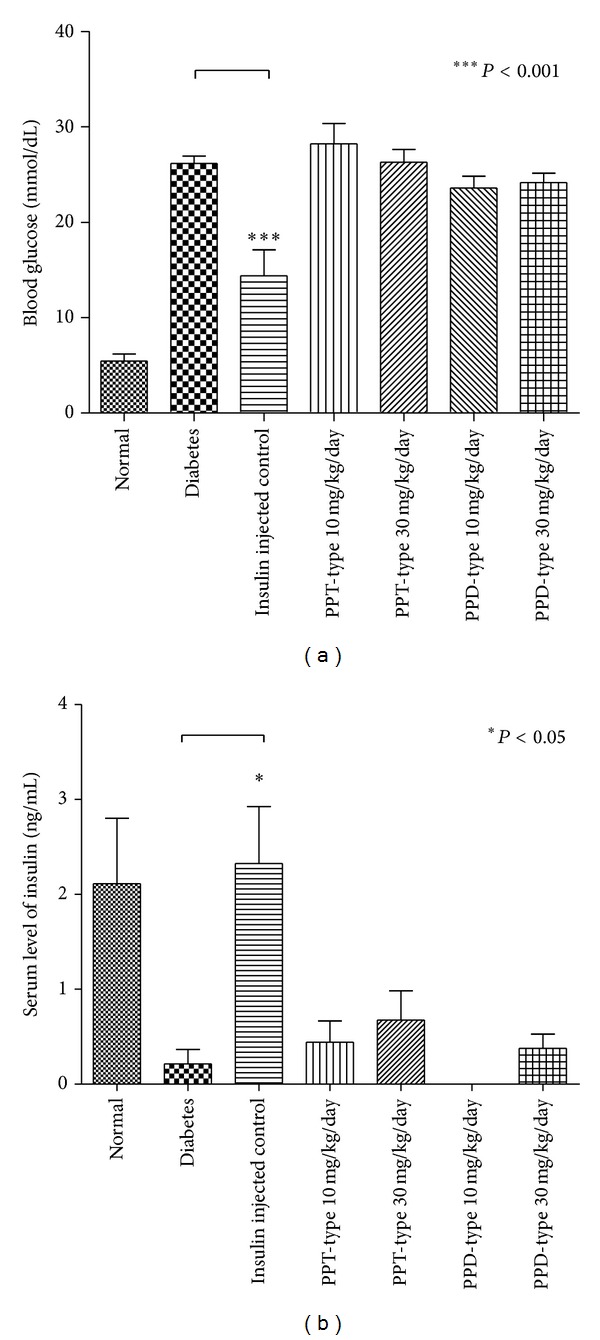
Blood glucose level (Panel (a)) and serum insulin level (Panel (b)) of control, diabetic, and ginseng extract-fed diabetic groups. The bar indicates standard error; *P < 0.05 for the indicated comparisons versus diabetic group.

**Figure 4:**
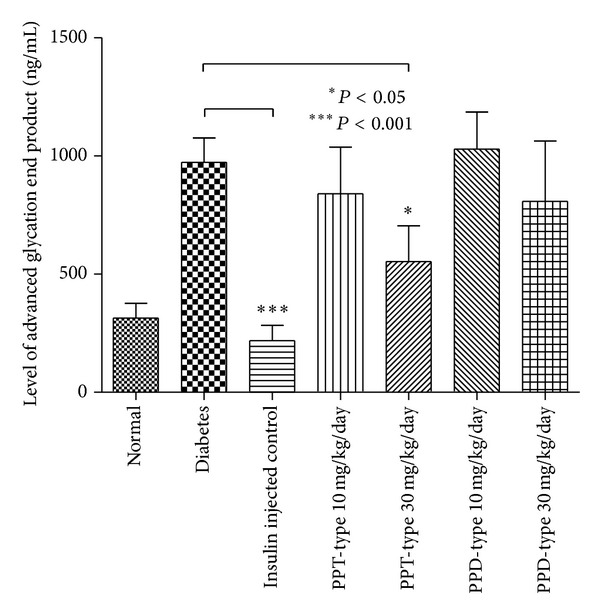
Level of advanced glycation end product in serum of control, diabetic, and ginseng extract-fed diabetic groups. Results were expressed as the mean ± standard error; *P < 0.05 for the indicated comparisons versus diabetic group.

**Figure 5:**
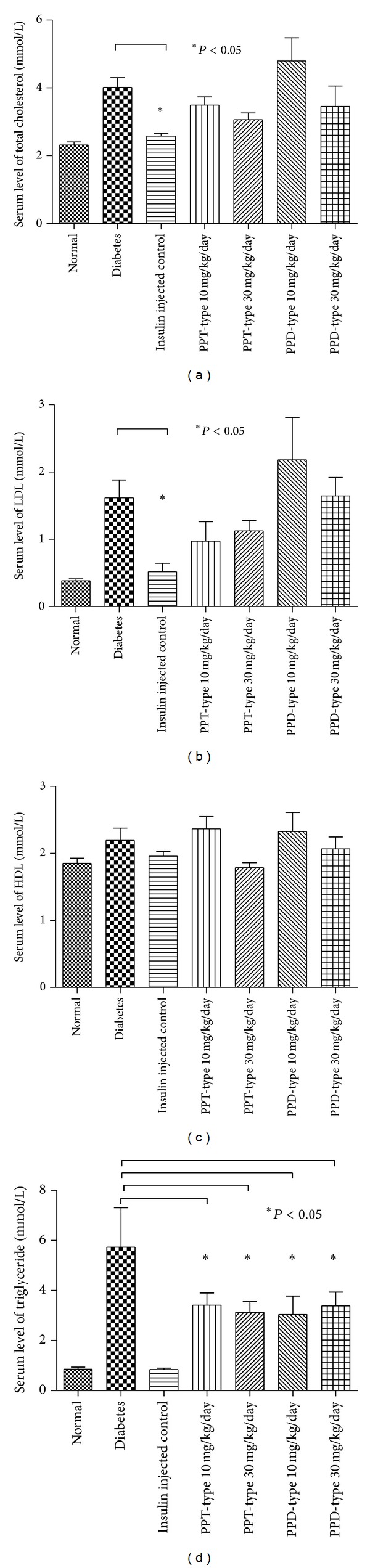
Serum levels of total cholesterol (Panel (a)), LDL (Panel (b)), HDL (Panel (c)), and triglyceride (Panel (d)) in control, diabetic, and ginseng extract-fed diabetic groups. Results were expressed as the mean ± standard error; *P < 0.05 for the indicated comparisons versus diabetic group.

**Figure 6:**
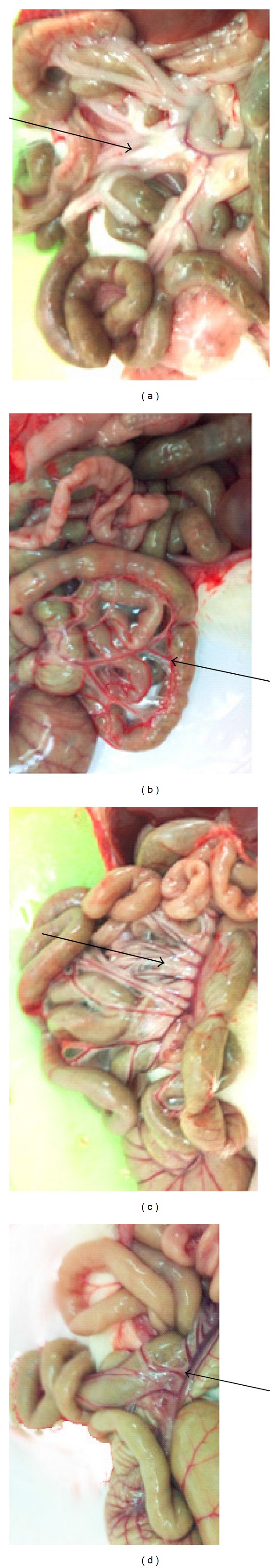
Distribution of visceral adipose tissue surrounding mesenteric arteries. The mesenteric bed from normal rats (Panel (a)) is surrounded by adipose tissue, whereas the mesenteric bed of diabetic rats (Panel (b)) is not surrounded by any adipose tissue. The PPD-type fed diabetic group (Panel (c)) and PPT-type fed diabetic group (Panel (d)) have comparatively more adipose tissue than the diabetic group.

**Figure 7:**
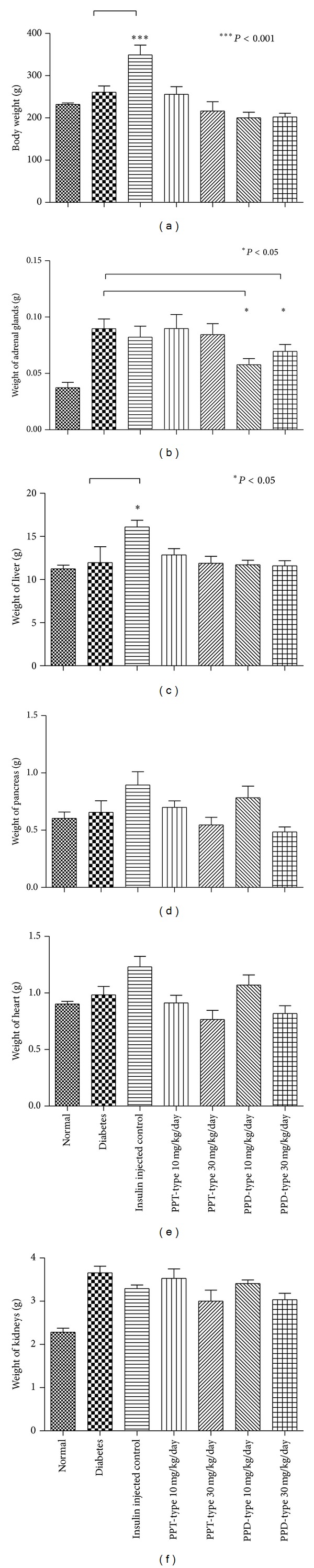
Weights of rats (Panel (a)) and weights of adrenal gland (Panel (b)), liver (Panel (c)), pancreas (Panel (d)), hearts (Panel (e)), and kidneys (Panel (f)). The insulin injected control group is slightly heavier than other groups. Results were expressed as the mean ± standard error; *P < 0.05 for the indicated comparisons versus diabetic group.

In this study, blood glucose, insulin, advanced glycation end products, serum total cholesterol, high density lipoprotein (HDL), low density lipoprotein (LDL), and triglyceride were examined. Except for normal and insulin injected positive control group, all of the diabetic groups were considered to be diabetic (blood glucose >16.7 mmol/dL) and with a significant reduction of insulin level (Figure 3(a)). There are no statistical differences between the ginseng-fed or nonfed diabetic groups for blood glucose, insulin, serum total cholesterol, HDL, and LDL (Figures 3 and 5(a)–5(c)), indicating the ginseng extracts have no improvement on hyperglycemic conditions or alternation of cholesterol levels. However, there is a slight decrease in the level of glycation end products (Figure 4), when the diabetic group was fed with PPT-type of ginseng at a dosage of 30 mg/kg/day. In addition, there was also significant decrease in serum triglyceride level for all ginseng-fed groups, which indicated that both PPD-type and PPT-type are effective in lowering serum triglyceride (Figure 5(d)). Visceral adipose tissue is associated with fatty acid metabolism. The distribution of visceral adipose tissue surrounding mesenteric arteries was shown in Figure 6. More visceral adipose tissue was found in control group when compared to the diabetic group. However, more visceral adipose tissue was observed in diabetic rats after feeding with PPD-type and PPT-type of ginseng extracts. The body mass and organ mass are the health indicators for the experimental rats.  Figure 7 showed the body and organ weight of the experimental rats. The body weight of the insulin-injected diabetic groups is slightly larger than other groups. Among all organs measured (liver, pancreas, heart, adrenal gland, and kidneys), the PPD-type fed diabetic groups have significantly smaller adrenal glands than diabetic group (*P* < 0.05).

### 3.3. Ginseng Extract Suppresses the Expression of Atherosclerosis-Related Genes

**Figure 8:**
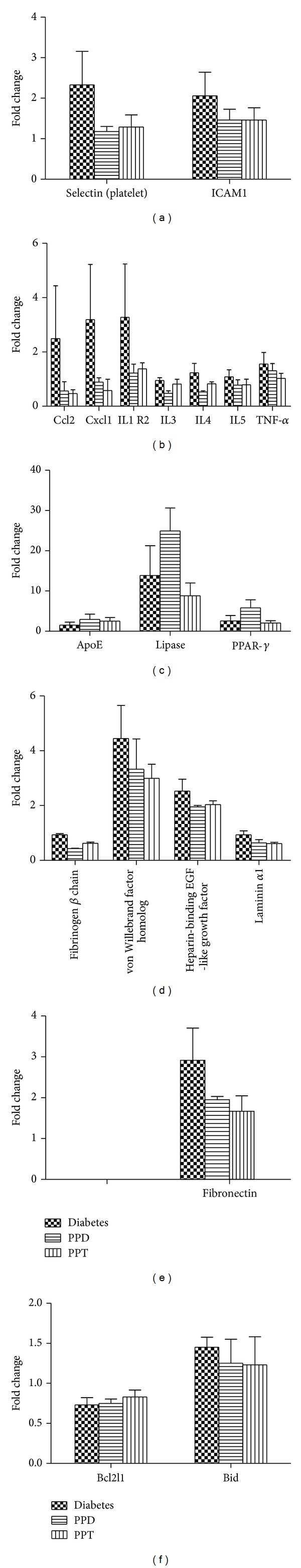
Comparison of different atherosclerosis related-gene expressions on adhesion molecules (Panel (a)), macrophages (Panel (b)), lipid metabolism (Panel (c)), smooth muscle cells proliferation and migration (Panel (d)), extracellular matrix (Panel (e)), and apoptosis (Panel (f)) by PCR array analysis. The PPD and PPT groups were fed with PPD-type and PPT-type of ginseng extract at dosage of 30/mg/kg/day, respectively. The fold change for normal control was set at 1.

PCR array analysis showed the fold change of atherosclerosis-related gene expression (Figure 8 and Table 2) for different treatment groups. When compared to normal control group, diabetic groups showed an upregulation on several atherosclerosis-related gene expressions, which indicate an increased risk of atherosclerosis. Besides, the gene expressions related to inflammations including adhesion molecules such as selectin (platelet) and ICAM1 and macrophage activation including chemokine (C-C) motif ligand 2 (CCl-2), chemokine (C-X-C) motif ligand 1 (CxCl-1), interleukin 1 receptor 2 (IL1-R2), interleukins (IL3, IL4 and IL5), and tumor necrosis factor-*α* (TNF-*α*) were downregulated. Apart from genes related to inflammation, other genes involved in the development of atherosclerosis were also checked. Apoptotic genes, such as Bid as well as genes responsible for vascular endothelial cells and vascular smooth muscle cell proliferation and migration (including von Willebrand factor homolog, heparin-binding EGF-like growth factor, and thrombospondin 4), were also downregulated in ginseng-fed diabetic groups. On the other hand, lipid-related genes expression including apolipoprotein E (ApoE), lipase, and peroxisome proliferator-activated receptor (PPAR) *γ* were increased in the ginseng-fed groups.

In general, the ginseng-fed diabetic groups showed a decreased expression on atherosclerosis-related genes, which indicates the decreased risk of atherosclerosis after ginseng treatments. 

## 4. Discussion

Endothelium controls vascular tone through the production of vasodilator mediators, endothelium-derived relaxing factors (EDRF), which act on vascular smooth muscle cells. The EDRF comprise nitric oxide (NO), prostacyclin, and an elusive endothelium-derived hyperpolarizing factor (EDHF). Multiple mechanisms lead to endothelial dysfunction [11, 12], and endothelial dysfunction plays a key role in the pathogenesis of vascular diseases. Hyperglycemia is linked to the pathogenesis of diabetic complications involving alternations of intracellular metabolism and formation of advanced glycation end products.

The attenuated endothelium-dependent vasodilations have been demonstrated in various vascular tissues of diabetic animal model [13]. In the present study, we examined the endothelial functions using the physiological isolated tissue bath setup and found that the high glucose-impaired vasodilations were restored after ginseng extracts treatment (Figure 2). The result indicates that ginseng extract plays a protective role in restoring normal endothelial functions in diabetic models. Different molecular mechanisms have been demonstrated to cause the vascular dysfunctions. Reports have suggested that hyperglycaemia-induced endothelial dysfunction is due to activation of protein kinase C (PKC) [14], inhibition of endothelial nitric oxide synthase [15, 16], early and advanced nonenzymatic glycation, and oxidative stress [17–19]. In atherosclerotic conditions, up-regulation of adhesion molecules, increased cytokine secretion, apoptosis, enhanced low-density lipoprotein oxidation, platelet activation, and vascular smooth muscle cell proliferation and migration are always observed [20–22]. Therefore, compounds that are able to modulate atherosclerosis and maintain normal endothelial functions are highly desirable. 

Although the levels of LDL, TC, HDL, and insulin in diabetic group were not significantly different when compared with the ginseng-fed groups, ginseng-fed diabetic groups showed a decrease in serum triglyceride level after ginseng feeding (Figure 5(d)). Increased levels of serum triglyceride and free fatty acids are common features of diabetic dyslipidemia [23]. There are direct corelations of serum triglyceride with triglyceride-associated nonalcoholic fatty liver disease (NAFLD), which is a multifactorial syndrome linked with cardiovascular diseases [24]. To further investigate the underlying mechanisms of the altered triglyceride metabolism, we performed PCR array analysis to examine the changes of gene expressions in rat aorta after ginseng treatment. By comparing normal, diabetic, and ginseng-fed diabetic groups, we have studied the change of expression in 87 different atherosclerosis or lipid metabolism related genes. Several lipid metabolism related genes such as ApoE, lipase, and PPAR-*γ* are upregulated in the aorta of ginseng extract-fed groups when compared to diabetic control group, showing the beneficial effects of ginseng. ApoE is responsible for catabolism of triglyceride-rich lipoprotein and cardiovascular diseases and was found to be related to proinflammatory cytokines [25]. On the other hand, up-regulated gene expression of lipase leads to increase process of dietary lipids (e.g., triglyceride) which may explain the decreased triglyceride levels. PPAR-*γ* is up-regulated by PPD, and it is the target of thiazolidinediones, the drugs used in treatment of diabetes mellitus. The upregulations of these genes provide possible explanation to the lowered triglyceride levels.

There is no statistically significant difference in body weight among the normal and the diabetic groups, possibly due to large variations of body weights of diabetic groups. Interestingly, the insulin-injected diabetic control group has significant weight gain (Figure 7(a)). The weight gain in the insulin-injected diabetic group has been reported by Jansen et al. in 2010 [26], which may be due to insulin therapy. The weight of adrenal glands (Figure 7(b)) is significantly smaller in the groups fed with PPD-type groups of ginseng extract except the liver (Figure 7(c)); other organs including pancreas, heart, and kidneys are not significantly different in weight among all diabetic groups (Figures 7(d)–7(f)).

Known to be responsible for “fight-or-flight” response, the size of adrenal glands reflects adrenocorticoid secretion [27], and adrenal enlargement is directly related to stress [28] like diabetes mellitus [29]. Interestingly, though insulin therapy is the known most effective method for diabetes, it cannot reverse adrenal gland enlargement. This may due to intensive injection of insulin which imposed stress on the rats. However, PPD-type extract, at both dosages of 10 mg/kg/day and 30 mg/kg/day, can reduce the size of enlarged adrenal glands in diabetic groups significantly (Figure 7(b)).

It has been shown with evidence that endothelial apoptosis might be a major cause of plaque erosion [30]. If there is endothelial apoptosis, lipid-laden foam cells derived from macrophages produce phospholipid oxidation products (OX-PL) and play a role in atherosclerosis. There are two forms of atherosclerotic plaques, (1) stable plaque, which is made up of thick fibrous cap isolating small lipid core and associated with low risk of thromboembolic complications, and (2) unstable plaque, which is a large lipid core covered by thin fibrous cap and prone to rupture and thrombus formation and associated with high risk of thromboembolic complications [31]. Hence, in addition to the decrease in triglyceride levels and changes in lipid metabolism related genes expression, the decrease in apoptosis-related genes such as Bcl2-like 1 and Bid may also help to reduce risk of atherosclerosis and restore normal aorta vasorelaxation. Figure 6 shows that there is more visceral adipose tissue in the ginseng-fed groups, and the observation may be related to the altered lipid metabolism in diabetic conditions. It is known that visceral adipose tissue is linked to fatty acid metabolism [32]. However, as most of the researches focus on the adverse effects of visceral adipose tissue which is a common observation in obesity, we cannot find any evidence to explain the current phenomenon. However, it has been found that ginsenoside Rb1 promotes adipogenesis in 3T3-L1 cells by enhancing PPAR-*γ*2 and C/EBP-*α* functions [33]. According to our present data on increased PPAR-*γ* expression, we believe that ginseng extract can modulate lipid metabolism in the diabetic condition through altered gene expression, which may result in an increased amount of visceral adipose tissue. 

 Furthermore, similar observation has been reported by a recent paper published by Liu et al. [34], who showed that ginsenosides can lower both triglyceride and total cholesterol level. In contrast, our results demonstrated a decrease in only the triglyceride level by using a lower dosage of ginseng extract. Therefore, we believe that ginseng may have potential therapeutic effects on elevated lipid levels and this effect may be dose dependent. 

Although glucose level is not lowered in all ginseng-fed diabetic groups, PPT-type ginseng extract fed at a dose of 30 mg/kg/day decreased the level of glycation end products in diabetic rats (Figure 4). Glycation end-product can be formed exogenously by heating or cooking or endogenously through normal aging or accelerated formation under diabetic conditions. The glycation process yields two different products: early and advanced glycation endproducts (AGEs). Recent finding shows advanced glycation end products formed on haemoglobin and HbA1c, which is a well-established important indicator for glycaemia monitoring. The advanced glycation end products that accumulate in vascular tissues are likely related to alterations in the connective tissue composition of the microvascular wall, which results in increased tissue rigidity [11]. During the pathogenesis of diabetes, endothelial cells intake more glucose [35, 36] and in turn increase the proton gradient and eventually produce reactive oxygen species and damaged DNA and more glycation end products will be produced. In other words, lower levels of advanced glycation end products usually show less hyperglycemic damages.

Biomarkers for accurately predicting clinical outcome and assessing disease risk and progression would greatly facilitate cardiovascular disease diagnosis and therapy. Finding the right balance between safety and efficacy of therapeutic methods probably requires assessing a variety of anti-inflammatory mechanisms and so forth [37]. Figure 8 showed a panel of atherosclerosis genes (including genes related to adhesion molecules, inflammation, vascular cell proliferation, and migration) which are downregulated. The findings may bring beneficial therapeutic implications to the vascular complications in diabetes. However, the underlying mechanisms, for example, increased lipid metabolism but observable increased amount of visceral adipose tissue, remain to be determined.

## 5. Conclusion

Ancient pieces of literature *Shennong Ben Cao Jing* and *Kai Bao Ben Cao* have mentioned the potential antiobesity and antidiabetic effects of *Panax ginseng. *Here, our present study proves that the endothelium-dependent relaxation can be impaired by diabetes mellitus and the damage can be protected by feeding with ginseng extracts (both PPD-type and PPT-type). Furthermore, the PCR array result reveals that ginseng may exert endothelial protection effect by downregulating the gene expressions of adhesion molecules, inflammatory cytokines, and chemokines. The protective mechanisms may partially due to lowering of serum triglyceride levels or alternating atherosclerosis-related and lipid-related gene expressions, which may result in anti-inflammation and endothelial cell protection. Therefore, further studies would be required to differentiate the protective mechanisms by individual ginsenosides in the future.

## Figures and Tables

**Table 1:** Contents of ginsenosides in the prepared PPT-type and PPD-type ginsenosides.

Sample	Ginsenosides	Content (mg/g)	Percentage
PPT-type ginsenosides	Rg1	290.9	29.09%
	Re	339.6	33.96%
PPD-type ginsenosides	Rb1	246.3	24.63%
	Rc	231.30	23.13%
	Rb2	136.0	13.61%
	Rd	84.5	8.45%

**Table 2:** PCR array analysis of expression change in selected atherosclerosis-related genes.

Gene name	Fold change^#^		
	Diabetes	PPD-fed diabetic group (30 mg/kg/day)	PPT-fed diabetic group (30 mg/kg/day)
*Adhesion molecules *			
Selectin (platelet)	+2.33	+1.18	+1.29
Intercellular adhesion molecule 1	+2.06	+1.46	+1.46
*Macrophages *			
Chemokine (C-C) motif ligand 2	+2.49	+0.56	+0.47
Chemokine (C-X-C) motif ligand 1	+3.19	+0.89	+0.58
Interleukin 1 receptor, type II	+3.28	+1.22	+1.38
Interleukin 3	+0.95	+0.48	+0.82
Interleukin 4	+1.23	+0.53	+0.83
Interleukin 5	+1.08	+0.78	+0.79
Tumor necrosis factor-*α*	+1.56	+1.31	+1.03
*Lipid metabolism *			
Apolipoprotein E	+1.53	+2.95	+2.50
Lipase	+13.85	+24.93	+8.82
Peroxisome proliferator-activated receptor-*γ*	+2.54	+5.83	+2.06
*Cell growth and migration *			
Fibrinogen beta chain	+0.93	+0.43	+0.62
von Willebrand factor homolog	+4.45	+3.33	+2.99
Heparin-binding EGF-like growth factor	+2.53	+1.95	+2.04
Laminin *α* 1	+0.93	+0.64	+0.61
*Extracellular matrix (ECM) *			
Fibronectin	+2.91	+1.94	+1.66
*Apoptosis *			
Bcl2-like 1	+0.73	+0.75	+0.83
BH3 interacting domain death agonist	+1.45	+1.25	+1.23

^#^Fold changes (comparing to control group, fold change = 1) are calculated according to manufacturer's analysis software.
